# Coalescence dynamics of platinum group metal nanoparticles revealed by liquid-phase transmission electron microscopy

**DOI:** 10.1016/j.isci.2022.104699

**Published:** 2022-07-01

**Authors:** Joodeok Kim, Dohun Kang, Sungsu Kang, Byung Hyo Kim, Jungwon Park

**Affiliations:** 1 School of Chemical and Biological Engineering, Institute of Chemical Processes, Seoul National University, Seoul 08826, Republic of Korea; 2 Center for Nanoparticle Research, Institute of Basic Science (IBS), Seoul 08826, Republic of Korea; 3 Department of Organic Materials and Fiber Engineering, Soongsil University, Seoul 06978, Republic of Korea; 4 Institute of Engineering Research, College of Engineering, Seoul National University, Seoul 08826, Republic of Korea; 5 Advanced Institutes of Convergence Technology, Seoul National University, Gyeonggi-do 16229, Republic of Korea

**Keywords:** Materials science, Materials chemistry, Materials characterization

## Abstract

Coalescence, one of the major pathways observed in the growth of nanoparticles, affects the structural diversity of the synthesized nanoparticles in terms of sizes, shapes, and grain boundaries. As coalescence events occur transiently during the growth of nanoparticles and are associated with the interaction between nanoparticles, mechanistic understanding is challenging. The ideal platform to study coalescence events may require real-time tracking of nanoparticle growth trajectories with quantitative analysis for coalescence events. Herein, we track nanoparticle growth trajectories using liquid-cell transmission electron microscopy (LTEM) to investigate the role of coalescence in nanoparticle formation and their morphologies. By evaluating multiple coalescence events for different platinum group metals, we reveal that the surface energy and ligand binding energy determines the rate of the reshaping process and the resulting final morphology of coalesced nanoparticles. The coalescence mechanism, based on direct LTEM observation explains the structures of noble metal nanoparticles that emerge in colloidal synthesis.

## Highlights

• Image processing of *in situ* liquid cell TEM image
• Size-dependent coalescence behaviors of metal nanoparticles
• Different kinetics of Pt and Pd nanoparticles owing to their different surface energies
• Reshaping kinetics determines the final morphology of coalesced nanoparticles

## Graphical abstract

**Figure:**
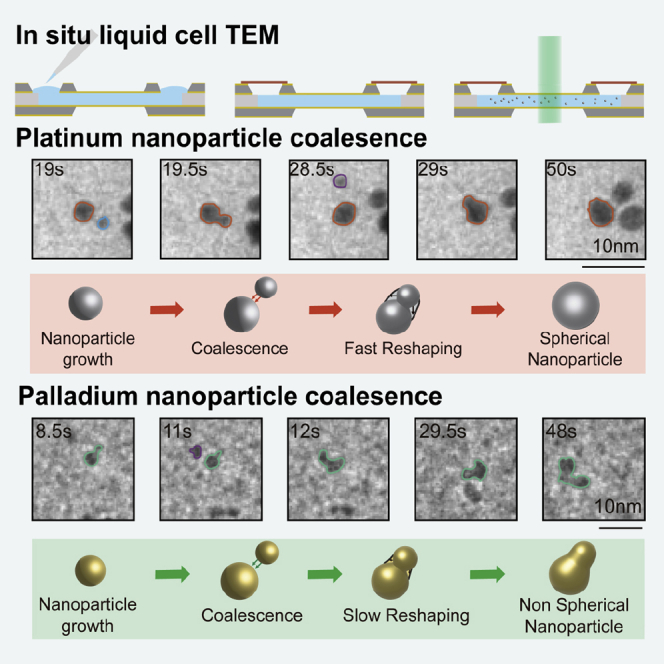

## Introduction

Understanding the formation mechanism of nanoparticles is critical for designing controlled synthesis of nanoparticles (Chang et al., 2019; Polte et al., 2010a, 2010b; Selvam and Chi, 2011). Conventionally, the formation of colloidal nanoparticles has been understood based on classical crystallization theory characterized by nucleation during the initial period of synthesis and subsequent growth driven by monomer attachment (Kwon and Hyeon, 2011; Talapin et al., 2001). However, in a typical size regime where nanoparticles undergo these processes during the synthesis, their surface-to-volume ratios are significantly high enough to make the surface of nanoparticles reactive (Ivanova and Zamborini, 2010; Jia and Schüth, 2011). Such reactivity provides possibilities for alternative pathways to be involved during monomeric growth (Lee et al., 2016; Wang et al., 2014). Coalescence, which denotes the attachment between two or more particles, is one of these non-classical pathways, while evidence of its mechanism has been observed in experiments using small-angle X-ray scattering (Ingham et al., 2011) and *in situ* X-ray absorption fine structure spectroscopy (Harada and Kamigaito, 2012). These experiments concurrently indicate that the coalescence of growing nanoparticles occurs along with the monomeric growth process. However, these studies are limited in that they neither identify the exact moments of coalescence events nor elucidate the effect of coalescence on the final structures of synthesized colloidal nanoparticles.

Direct observation of coalescence events at the level of individual nanoparticles was recently accomplished by *in situ* liquid-cell transmission electron microscopy (TEM) of colloidal nanoparticle growth (Anand et al., 2016; Chen et al., 2020; Yang et al., 2019; Yuk et al., 2012). In this approach, liquid cells encapsulate a specimen in the liquid phase and seal it against the high vacuum of TEM, allowing for real-time measurement with nanometer-scale resolution (Kim et al., 2018). Using this method, coalescence events were observed to frequently occur along with classical growth by monomer attachment, and both pathways arrive at a uniform final size determined by thermodynamic factors in the solution (Zheng et al., 2009). In addition, *in situ* liquid-cell TEM observations revealed that iron oxyhydride nanoparticles rotate to obtain an energetically preferred lattice plane before coalescence (Li et al., 2012; Liu et al., 2020; Zhu et al., 2018). The effects of ligands and solvent molecules, and the process of neck formation within a nanoparticle pair during coalescence were also examined via liquid-cell TEM (Jin et al., 2018; Lim et al., 2020). The surface-to-volume ratios of nanoparticles are generally high, rendering the achievement of sufficient driving force for coalescence possible. Such events are supposedly ubiquitous in colloidal solutions of nanoparticles (Zheng et al., 2009). The surface energy of nanoparticles, which is determined by the surface-to-volume ratio, van der Waals interactions, and dipole moments can be an important factor in coalescence events because it is associated with the governing factor of nanoparticle interactions. Furthermore, surface ligands used to stabilize the high surface energy of the growing nanoparticles are also relevant to the coalescence event because they can be involved in nanoparticle interactions (Bae et al., 2020). The morphology of the coalesced particles is likely to be determined by such factors as well.

Here, using *in situ* liquid cell TEM, we investigate the coalescence-driven growth of nanoparticles with different metal compositions. The tracking of single nanoparticle trajectories and quantitative analysis of acquired trajectories confirm that the coalescence events and reshaping processes of platinum (Pt) and palladium (Pd) nanoparticles show different kinetics owing to their different surface and ligand binding energies. We also elucidate that different coalescence kinetics results in distinct final morphologies of the synthesized nanoparticles.

## Results

### Direct observation of coalescence process in platinum and palladium nanoparticle growth using *in situ* liquid phase transmission electron microscopy

**Figure 1:**
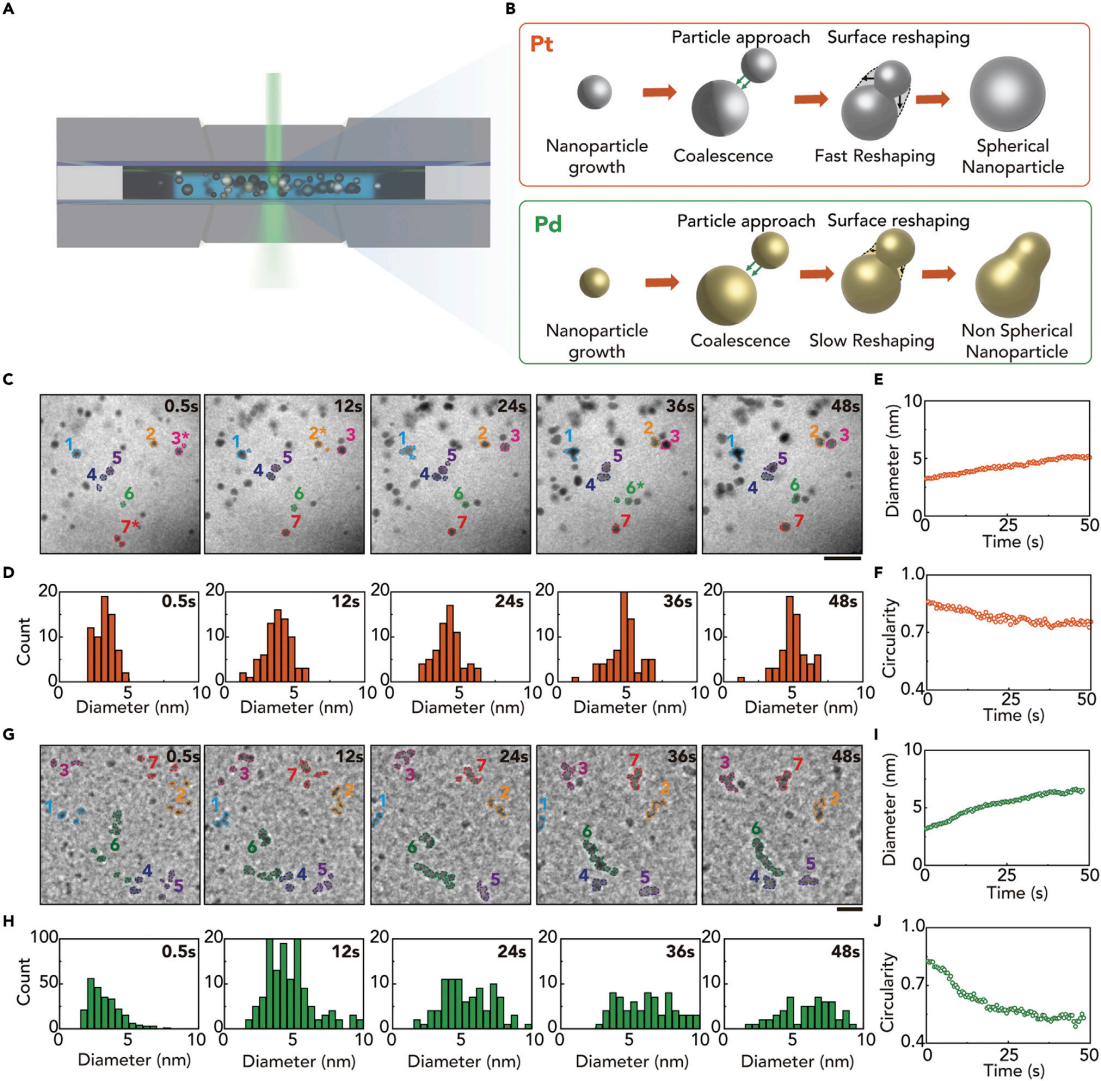
Observation of coalescence processes of Pt and Pd nanoparticles using liquid-phase TEM (A) Schematic diagram of a liquid cell TEM experiment for studying the coalescence of nanoparticles in a liquid cell TEM. (B) Schematic diagrams of the fast reshaping of coalesced Pt nanoparticles and slow reshaping of coalesced Pd nanoparticles. (C) Sequential TEM images of Pt nanoparticle growth in a liquid cell from Video S1. The nanoparticles that coalesce to form a single particle are marked with the same color. Scale bar: 10 nm. (D) Size histogram of tracked nanoparticles from the liquid-cell TEM images. (E) Average size and (F) average circularity of the Pt nanoparticles as a function of time. (G) Sequential TEM images of Pt nanoparticle growth in a liquid cell from Video S2. The nanoparticles that coalesce to form a single particle are marked with the same color. Scale bar: 10 nm. (H) Size histogram of tracked nanoparticles from the liquid-cell TEM images. (I) Average size and (J) average circularity of the Pt nanoparticles as a function of time.

We prepare a liquid cell compatible with normal TEM holders (Kim et al., 2017) for the *in situ* liquid-cell TEM experiment. The liquid cell comprises two 100-μm-thick cell bodies, two 50-nm-thick Si_3_N_4_ windows, and a 100-nm-thick spacer (Figure 1A). A precursor solution prepared by mixing 10 mg of metal precursor and 0.1 mL of oleylamine into 0.9 mL of dichlorobenzene solvent is loaded into the liquid cell. In this work, we compare the growth of two types of metal nanoparticles (Pt and Pd) using Pt(acac)2 and Pd(acac)_2_ as precursor solutions for the growth of Pt and Pd nanoparticles, respectively. In both systems, oleylamine works as a surface ligand. The nanoparticle growth is monitored using JEOL 2100 at an acceleration voltage of 200 kV. The *in situ* TEM images are obtained with a frame rate of two frames per second (Video S1. Pt nanoparticle growth with multiple coalescence events in the liquid cell TEM, related to Figure 1, Video S2. Pd nanoparticle growth with multiple coalescence events in the liquid cell TEM, related to Figure 1, Video S3. Pt nanoparticle growth with multiple coalescence events in the liquid cell TEM, related to Figure 5, Video S4. Pd nanoparticle growth with multiple coalescence events in the liquid cell TEM, related to Figure 5, Video S5. Pd nanoparticle growth with multiple coalescence events in the liquid cell TEM at dose rate 100 e−Å−2s−1, related to Figures S6 and S7).

Video S1. Pt nanoparticle growth with multiple coalescence events in the liquid cell TEM, related to Figure 1

Video S2. Pd nanoparticle growth with multiple coalescence events in the liquid cell TEM, related to Figure 1

Video S3. Pt nanoparticle growth with multiple coalescence events in the liquid cell TEM, related to Figure 5

Video S4. Pd nanoparticle growth with multiple coalescence events in the liquid cell TEM, related to Figure 5

Video S5. Pd nanoparticle growth with multiple coalescence events in the liquid cell TEM at dose rate 100 e^−^Å^−2^s^−1^, related to Figures S6 and S7

Irradiation of the electron beam in LTEM induces nucleation and growth of Pt or Pd nanoparticles by the reduction of the Pt(acac)_2_ or Pd(acac)_2_ precursors, respectively, followed by coalescence-mediated growth. The liquid TEM images of the growth of Pt and Pd nanoparticles reveal their different coalescence behaviors (Figure 1B). The coalesced Pt nanoparticles undergo a fast reshaping process while coalesced Pd nanoparticles retain their snowman-shaped morphology for a prolonged time. To investigate the different growth processes between Pt and Pd nanoparticles, we track nanoparticle size and shape in each frame of the *in situ* liquid-cell TEM images (Figures 1C–1J). The size of the nanoparticle is defined as the equivalent diameter (*D*) calculated from the projected area in the TEM images using the following equation, *D* = $2\times \sqrt{A/\pi }$, where A denotes the projected area of the nanoparticle. The shape of the nanoparticle is quantified by the circularity (C) evaluated by the relationship, C = $4\pi A/P^{2}$, where A and P denote the projected area and the perimeter of the nanoparticle, respectively. The average size of both types of nanoparticles continuously increases within 50 s (from 3.2 to 5.1 nm for Pt and from 3.2 to 6.5 nm for Pd) (Figures 1E and 1I). During size growth, the circularity of Pt nanoparticles persists at high values, indicating that the coalesced nanoparticles rapidly transform into spherical shapes (Figure 1F). On the other hand, Pd nanoparticles show a different coalescence mechanism from Pt nanoparticles (Figures 1G–1J). The magnified TEM snap-shot images of Pd nanoparticles reveal that the attached nanoparticles do not reconstruct into spherical shapes, but rather retain their dumbbell shapes. When multiple particles are attached, they form an irregular worm-like shape. Owing to the irregularity of the coalesced Pd nanoparticles, size distribution is broadened during the growth of nanoparticles (Figures 1H and 1I). The retention of the worm-like shapes of Pd nanoparticles is confirmed by the low circularity of about 0.5.

### Quantitative analysis of coalescence dynamics in platinum nanoparticle growth by individual trajectories

**Figure 2:**
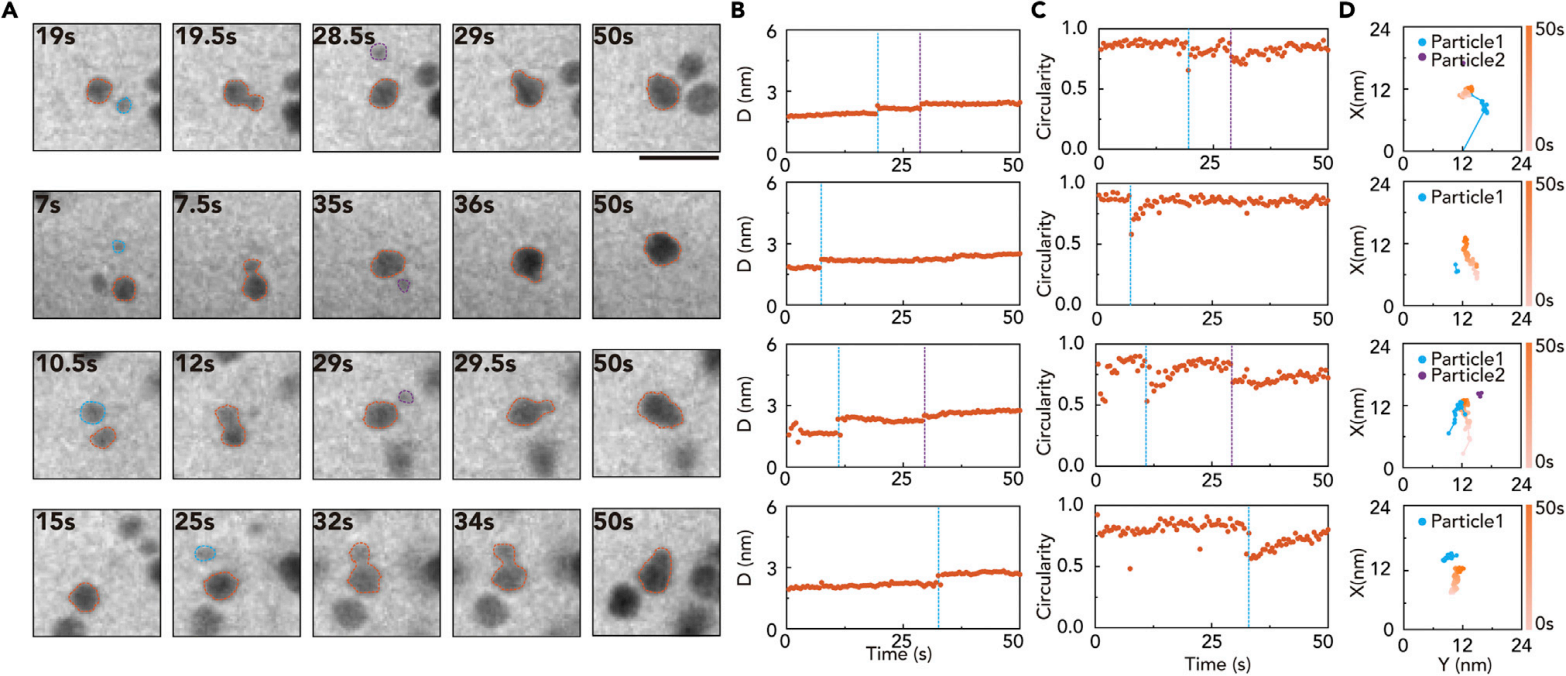
Coalescence dynamics of Pt nanoparticles (A) TEM snap-shot images for representative Pt nanoparticles that undergo multiple coalescence events. Scale bar: 10 nm. (B) Size change in the nanoparticle shown in panel (A). Dash lines indicate the time for coalescence events. (C and D) Circularity of nanoparticles shown in (A) with dashed lines (orange) and trajectories of each nanoparticle that undergoes multiple coalescence events.

We examine the individual trajectories of the merging nanoparticles to discover the detailed mechanism of the size and circularity change in the nanoparticles induced by the coalescence events. Four representative trajectories of Pt nanoparticle coalescence demonstrate the multiple coalescences in a single-nanoparticle growth trajectory (Figures 2A and 2B). Smaller nanoparticles are frequently attached to other particles which contribute to abrupt growth in size, while monomeric growth of nanoparticles simultaneously occurs as continuous growth. Interestingly, the trajectories and sizes of nanoparticles associated with coalescence (Figures 2C and 2D) indicate that the smaller nanoparticles are extremely mobile and rapidly coalesce into larger nanoparticles (Figures 2D and S1). The size narrowing is related to asymmetric coalescence. Small particles are consumed quickly, and the size distribution of particles can thus be narrow (Figure S2). Most coalescence events occur between 0 and 60 s, while this period overlaps the period during which the size distribution of the nanoparticles decreases (Figure S3).

We also observe the change in the shape of the nanoparticle during the coalescence and structural relaxation process. The dumbbell or snowman shapes formed after the coalescence can relax into a spherical structure (José-Yacamán et al., 2005; Wang et al., 2016), and the relaxation process is quantitatively investigated using the circularity change. The circularity of nanoparticles is maintained asymptotically close to 0.9, which value is close to a spherical shape. The value abruptly drops to approximately 0.5 at the initial stage of coalescence, for every coalescence step in multiple coalescence events. After the relaxation process, the circularity recovers to 0.8–0.9 within 10 s, indicating that the coalesced nanoparticles are rapidly reconstructed to spherical shapes (Grammatikopoulos et al., 2019; Hawa and Zachariah, 2006).

### Quantitative analysis of coalescence dynamics in palladium nanoparticle growth by individual trajectories

**Figure 3:**
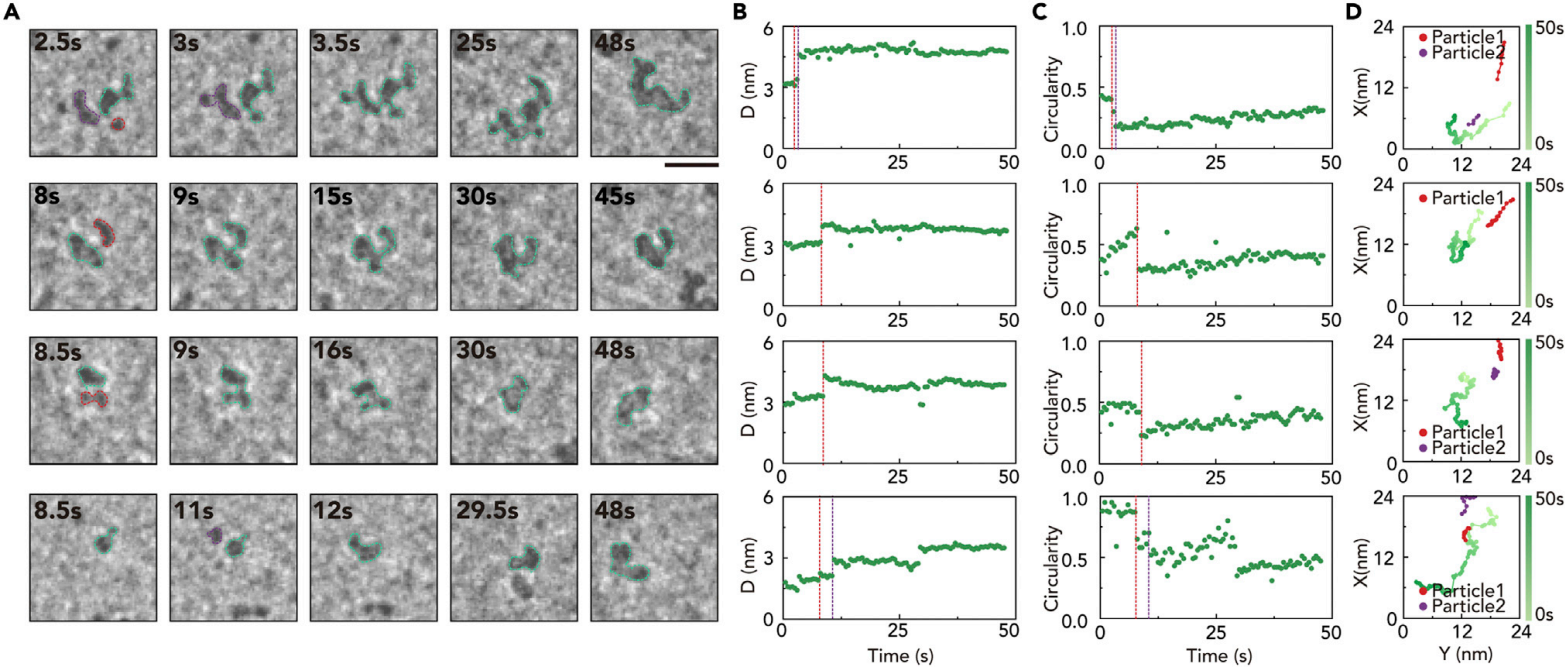
Coalescence dynamics of Pd nanoparticles (A) TEM snap-shot images for representative Pd nanoparticles that undergo multiple coalescence growth pathways. Scale bar: 10 nm. (B) Size change in the nanoparticle shown in panel (A). Dash lines indicate the time of coalescence events. (C and D) Circularity of nanoparticles shown in (A) with dashed lines (green) and trajectories of each nanoparticle that undergoes multiple coalescence events.

The Pd nanoparticles exhibit different coalescence dynamics from Pt nanoparticles. The *in situ* TEM images of individual Pd nanoparticles show that the coalesced nanoparticles form a snowman shape by the formation of the narrow neck between the two merging nanoparticles and retain this shape for a long time (Figures 3A and 3B). Although maintaining this irregular shape, additional nanoparticles are frequently attached to these snowman-shaped nanoparticles (Figures 3A and 3D). The combination of multiple coalescence and shape retention leads to worm-like shapes in the Pd nanoparticles. The intriguing shape changes are investigated by temporal changes in the circularity of individual Pd nanoparticles. As soon as two Pd nanoparticles are coalesced (red line in Figure 3C), the circularity drops by half. The reduced circularity is not restored to its initial value, but rather remains low, indicating that the shape is not reconstructed to a spherical shape. It is worth noting that the circularity value is less than 0.5 most of the time. Considering that the circularity of a square, an equilateral triangle, and a five-point star is 0.89 and 0.78, and 0.52, respectively (Olson, 2011), the low circularity means that the Pd nanoparticles have multiple concave parts as dumbbell, snowman, and worm-like shapes do.

### Different relaxation processes of the coalesced platinum and palladium nanoparticles

**Figure 4:**
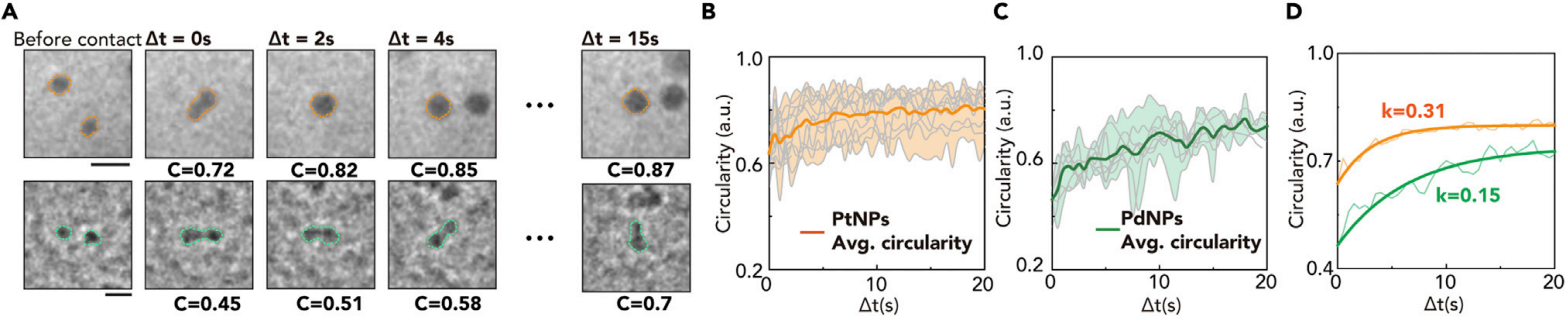
Relaxation process of the coalescence nanoparticles (A) TEM snap-shot images of representative Pt (top row and orange color) and Pd (bottom row and green color) nanoparticles that undergo a relaxation process after the coalescence event. Scale bar: 5 nm. (B and C) Circularity trajectories of merged 15 Pt and 8 Pd nanoparticles. The thick line indicates the change in the averaged circularity. Δ*t* is set to 0 for the time of surface contact. (D) Exponentially fitted averaged circularity change in the nanoparticle in (B) and (C), indicating that the relaxation rate constant of Pt (orange) is lower than Pd (green).

**Figure 5:**
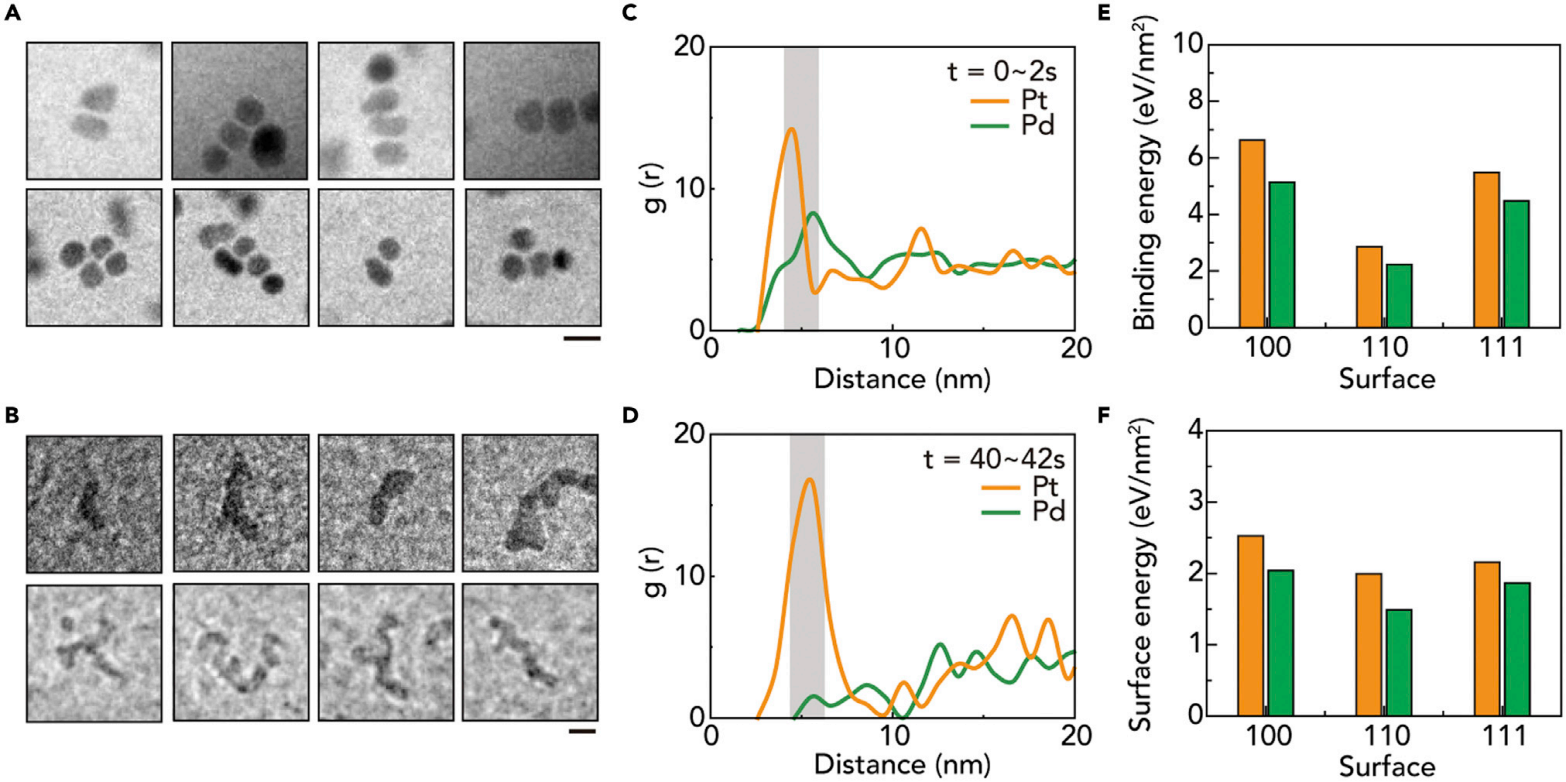
Different coalescence tendencies in the later stage of nanoparticle growth (A and B) TEM snap-shot images of representative nanoparticles from the later stage in (A) Pt growth (Videos S1 and S3) and (B) Pd growth (Videos S2 and S4). Scale bar: 10 nm. (C and D) Radial distribution function histogram of tracked nanoparticle for (C) early stage of growth (0∼2s) and (D) later stage of growth (40–42s). The histogram is binned by 1 nm for the distance between all particle pairs. (E and F) Calculated ligand binding energy and surface energy by the lowest configuration.

The relaxation process of the coalesced Pt and Pd nanoparticle is evaluated using the temporal circularity change after the coalescence event (Figure 4A). We select the coalescence processes among circular nanoparticles of similar sizes before their merging to minimize the influence of size and morphology before coalescence (15 pairs for Pt, 8 pairs for Pd) (Figures 4B and 4C). Figures 4B and 4C show the entire tracked circularity change in multiple Pt and Pd nanoparticles by setting *t*_0_ for the time of surface contact. For Pt nanoparticles, the circularity drops to about 0.65 at the initial stage of coalescence and recovers to about 0.8 during the relaxation process. In contrast, the circularity of Pd nanoparticles drops to about 0.5 and then slowly recovers to about 0.7. To quantitatively investigate the relaxation process after coalescing, we calculate the rate constant (*k*) of the relaxation process based on the simple model where the deviation of the circularity of the merged particle from the perfect spherical shape (circularity = 1) decays exponentially (Bae et al., 2020). The relaxation process is fitted based on the change in the circularity (C) over time. The difference between the C and C_*f*_. (circularity when relaxation process ends) decays exponentially, $C_{f}\;-\;C\left(\Delta t\right)\;=\;C\left(t_{0}\;\right)\times \;e^{-k\Delta t}$, where *t* is time. By fitting averaged circularity changes for the two cases, the rate constant of coalesced Pd nanoparticles is 0.15 s^−1^ while that of Pt is 0.31 s^−1^ (Table S1). The rate constant for merged Pt nanoparticles is two times higher than that of Pd nanoparticles, indicating that the relaxation process of a coalesced Pd nanoparticle happens much slower than for a Pt nanoparticle. The relaxation rate is different possibly because the surface energy of Pt is higher than that of Pd, which is calculated by density functional theory (Figures 5E and 5F). Owing to the high surface energy of Pt, the Pt nanoparticles prefer to reduce their surface-to-volume ratio, resulting in the relaxation process. On the other hand, Pd nanoparticles with low surface energy require a longer time to be relaxed to a spherical shape. Interestingly, the size ratio between the two approaching nanoparticles does not affect the rate constant of the relaxation process (Figure S4). In addition, Pt nanoparticles can remain circular despite multiple coalescences. As nanoparticles that do not undergo the coalescence process maintain their circular shape during the observation time, morphological changes occur only during the coalescence process (Figure S5). Owing to the differences in the relaxation rates of Pd and Pt nanoparticles based on the surface energy differences, nanoparticles synthesized from the two types of platinum group metal precursors exhibit different morphologies. We conduct the control experiment with the low electron beam dose rate (Figures S6, S7, and Video S5). Coalescence behaviors of nanoparticles with different dose rates show similar behaviors in terms of the slow relaxation process, worm-like morphology, and low circularity value after coalescence, indicating that coalescence events of nanoparticles observed in LPTEM are not strongly influenced by the dose rate.

### Different coalescence tendencies in the later stage of nanoparticle growth owing to the influence of surface and ligand binding energy

In the later stages of nanoparticle growth, Pt and Pd nanoparticles exhibit different behaviors in inter-particle interactions. For Pt nanoparticles, instead of coalescence that mainly occurs in the earlier stage, two approaching nanoparticles form a nanoparticle pair without merging events by maintaining a persistent gap between each other (Figure 5A). On the contrary, Pd nanoparticles still undergo coalescence with approaching nanoparticles (Figure 5B), forming irregular worm-like shaped nanoparticles in the late stage of growth. To quantitatively evaluate this trend, we calculate the radial distribution function (RDF) using the tracked nanoparticle trajectories (Lee et al., 2017; Liu et al., 2020). RDF is defined by g(r) = $\frac{1}{\pi Nr\rho_{0}}\sum_{j=1}^{N}\sum_{i>j}^{N}\left(r-r_{ij}\right)$, and represents the density of nanoparticles as a function of distance from a particle. At the initial stage of nanoparticle growth (at 0 to 2 s), the RDF does not show a significant difference between the Pt and Pd nanoparticles, and the most probable peaks are similar between the two systems (Figure 5C). In the later stage of Pt nanoparticle growth (at 40 to 42 s), the most probable peak is concentrated around 6 nm, which corresponds to the sum of the radii of two nanoparticles and the length of the ligand (Figure 5D). This result suggests that the approaching Pt nanoparticles do not coalesce anymore but assemble with ligand inter-digitation. The prevention of coalescence for these large-sized Pt nanoparticles that appeared in the later stage of nanoparticle growth can be explained in terms of surface energy and activation energy. After sufficient growth of Pt nanoparticles, the surface energy of the nanoparticle decreases owing to the reduced surface-to-volume ratio, thereby preventing coalescence events. The absence of coalescence events for the large nanoparticles with high curvature can be also explained by high coalescence activation energy, which is mainly attributed to difficult ligand displacement in large-sized nanoparticles with large curvature. The high activation energy prevents approaching nanoparticles from coalescing with each other (Grammatikopoulos et al., 2019). However, in the case of Pd nanoparticles (Figure 5E), the nanoparticles cannot maintain the gap between each other, so the RDF peak disappears in the small radius. As the surface binding energy of the ligand is low, the energy barrier of the coalescence process of the Pd nanoparticle is lower than that of the Pt nanoparticle (Figure 5F), and nearby particles easily coalesce into the larger nanoparticle. Moreover, the small curvature relative to size, which is attributed to the worm-shaped nature reduces the activation energy of coalescence and allows the coalescence event to occur, even at large sizes. The difference between surface energy and coalescence activation energy of Pt and Pd affects the relaxation process after the coalescence. The unique coalescence dynamics are supported by the previous study for ligand-dependent coalescence (Bae et al., 2020). In the literature, the low binding energy of ligands lowers the energy barrier of the coalescence process, which makes it difficult to create a gap between nanoparticles, leading to the synthesis of irregular worm-like shaped nanoparticles.

## Discussion

In this study, we systematically investigate coalescence dynamics of platinum group (Pt and Pd) metal nanoparticles using liquid-cell TEM. The analysis of the nanoparticle growth trajectories shows that nanoparticle growth is affected by frequent coalescence events. The coalesced Pt nanoparticles rapidly relax to spherical shapes while coalesced Pd nanoparticles retain their snowman shapes owing to the retarded relaxation process. In the late stage of Pd nanoparticle growth, multiple coalescence events and the retention of shape lead to the formation of worm-like shapes. On the other hand, already-grown Pt nanoparticles are assembled with ligand inter-digitation without coalescence process owing to their high activation energies attributed to their large curvature and high surface energy. Our studies suggest that the coalescence process regulates the shapes and structures of the synthesized nanoparticles.

### Limitations of the study

This research proposed the role of a binding process in nanoparticle growth mechanism and provides the perspective regarding morphological differences observed in the synthesis of metal nanoparticles with different compositions. This study is based on the real-time tracking of nanoparticle growth by LTEM. However, the measurements with low-resolution LTEM only allow understanding of processes based on morphological changes of nanoparticles. If this study were to be conducted with high-resolution LTEM, kinetics associated with coalescence events could be understood at a level that incorporates information on crystal structures and orientations of interacting nanoparticles. Other factors that may affect the change in surface energy and inter-particle interaction can be considered to extend understanding the underlying mechanism behind nanoparticle coalescence. The coalescence behaviors can be modulated by changing ligand species of nanoparticles (Bae et al., 2020) and liquid solvent-ligand interaction that modifies the surface energy. In addition, dipole-dipole interaction (Liu et al., 2020), lattice direction (Li et al., 2012), particle-surface interaction (Lu et al., 2014), van der Waals forces, and liquid film thickness (Kang et al., 2021) affect the particle-particle interaction for the nanoparticle coalescence and relaxation process.

## STAR★Methods

### Key resources table

**Table:** 

REAGENT or RESOURCE	SOURCE	IDENTIFIER
**Chemicals, peptides, and recombinant proteins**		

olyelamine, 70%	Sigma-Aldrich	Cat#O7805
Platinum acetylacetonate 97%	Sigma-Aldrich	Cat#282782
Palladium acetyl acetonate 99%	Sigma-Aldrich	Cat#209015
1,2-dichlorobenzene,>99%	TCI	D1116
Acetone 99.8%	Daejung Chem.	1009–2304
Pottasium hydroxide, 95%	Samchun Chem.	P0925
p-type silicon-on-insulator wafers	Soitec	Power-SOI
AZ 5214 E	AZ Electronic Materials	AZ 5214 E
AZ 327	AZ Electronic Materials	AZ 327
Indium pellets 99.98–9.99%	Kurt J. Lesker Company	EVMIN40EXEB

**Software and algorithms**		

MATLAB R2020a	The Math Works, Inc.	https://www.mathworks.com/
ImageJ	Schneider et al. (2012)	https://imagej.nih.gov/ij/

**Other**		

JEOL 2100F Field Emission Electron Microscope	JEOL	JEM-2100F

### Resource availability

#### Lead contact

Further information and requests for resources and data should be directed to and will be fulfilled by the lead contact, Jungwon Park (jungwonpark@snu.ac.kr)

#### Materials availability

This study did not generate new unique reagents.

### Method details

#### Acquisition of TEM images

TEM observations of the metal nanoparticle growth in the liquid cell were performed on a JEM-2100F (JEOL, Japan) instrument, operated at 200 kV, and equipped with an Ultra-Scan 1000XP CCD detector (Gatan). Further, *in situ* TEM movies were recorded in 2fps due to the high signal-to-noise ratio. The dose rate of the electron-beam was consistently maintained, the dose rate of the electron-beam was maintained around 1000 e^−^Å^−2^s^−1^ for Videos S1–S3, for Video S4, was 8000 e^−^Å^−2^s^−1^, for Video S5. Was 100 e^−^Å^−2^s^−1^. The *in situ* TEM movies were recorded in 2fps.

#### Particle tracking and evaluation

For analyzing *in situ* TEM images, we used homemade MATLAB code to evaluate nanoparticle size and morphology. Our code was developed for reducing the Gaussian noise, employing Gaussian and Weiner filters, and thereafter, a high-contrast change, at the edges of the nanoparticles, were emphasized through Laplacian filtering. Thereafter, the images were binarized by the adaptive thresholding method. We selected the dark areas to represent the nanoparticles and tracked the nanoparticles, which underwent coalescences. Particle tracking was conducted by particle distance between frame to frame.

We measured parameters for all the tracked nanoparticles from the time series of binarized images. For the measurement of the binarized image, we used MATLAB built-in regionprops function for measuring the area, centroid, and perimeter of nanoparticles.

#### DFT calculation of surface and ligand binding energy

We utilized first-principles density functional theory (DFT) calculations as implemented in Vienna ab-initio simulation package (VASP) (Kresse, 1994; Kresse & Furthmüller, 1996a, 1996b; Shimojo et al., 2001) with Perdew-Burke Ernzerhof (PBE) generalized gradient approximation (GGA) (Perdew et al., 1996) exchange-correlation functional and projector-augmented wave (PAW) (Blochl, 1994; Kresse and Joubert, 1999) pseudopotentials. Basic plane waves were expanded with a cutoff energy of 400 eV. We included the spin polarization correction and van der Waals (vdW) interaction with the DFT-D3 method by Grimme (2006).

The vacuum space was imposed to secure 10 Å within periodic images for each system. Monkhorst-Pack k-point meshes for the calculation of surface energies of (100), (110), and (111) surfaces are assigned as 7 × 7 × 1, 5 × 7 × 1, and 7 × 7 × 1, respectively. The surface energy (σ) was calculated with the equation below.

(Equation 1) $$\sigma =\;\frac{1}{2A}\;\left(E_{surf}-N_{atoms}\text{Δ}E_{bulk}\right)$$,

where $E_{surf}$ is the energy of the relaxed surface slab, $E_{bulk}$ is the energy of the bulk, $N_{atoms}$ is the number of atoms on the surface, and A is the area of one side of the surface.

For the calculation of ligand binding energies, the ligand, oleylamine is simplified as ethylamine since it is known to well represent its electronic properties. The optimal surface adsorption density on Pt (100), Pt (110), Pt (111), Pd (100), Pd (110), and Pd (111) was 4.32 eV/nm^2^, 2.29 eV/nm^2^, 3.75 eV/nm^2^, 4.41 eV/nm^2^, 2.24 eV/nm^2^, 3.82 eV/nm^2^, with calculated binding energies(*E_ads_*) by following equation, respectively:

(Equation 2) $$E_{ads}=E_{*ligand}–\left(E_{surface}+E_{ligand}\right)$$,

where $E_{*ligand}$ is the energy of the relaxed ligand bond surface slab, and $E_{ligand}$ is the energy of ligand (Figure S8).

## Data Availability

All data reported in this paper will be shared by the lead contact upon request. There is no original code associated with this work. Particle size and position, circularity and original movie.
