# Supplementary material for: Coalescence dynamics of platinum group metal nanoparticles revealed by liquid-phase transmission electron microscopy
Source: iScience. 2022 Jul 1;25(8):104699. doi: 10.1016/j.isci.2022.104699 (PMC9307684; doi:10.1016/j.isci.2022.104699)
Supplement: Document S1. Figures S1–S8 and Table S1 [file mmc1.pdf]

iScience, Volume *25*

Supplemental information

Coalescence dynamics of platinum group metal nanoparticles revealed by liquid-phase transmission electron microscopy

Joodeok Kim, Dohun Kang, Sungsu Kang, Byung Hyo Kim, and Jungwon Park

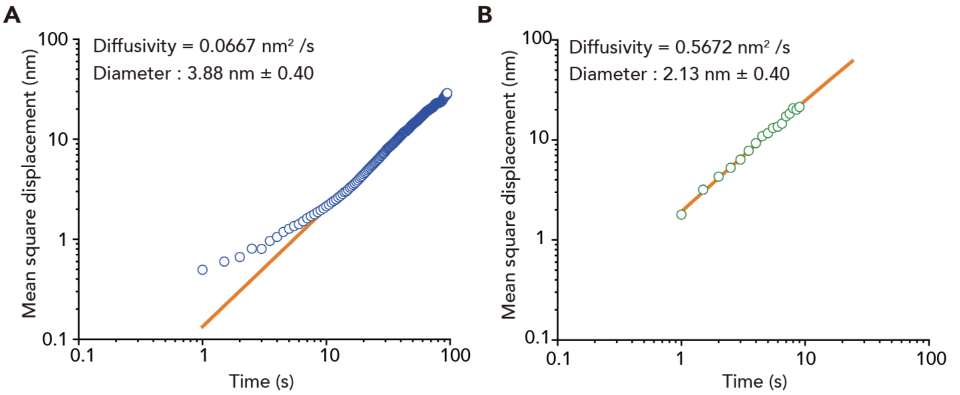

Supplemental Figure 1. MSD plot of representative nanoparticles with different sized nanoparticles. Related to Figure 2.

(A) MSD plot of representative nanoparticles with large-sized nanoparticles with an average diameter of 3.88 nm, showing relatively low diffusivity (D = 0.0667 nm^2/s).

(B) MSD plot of representative nanoparticles with small-sized nanoparticles with an average diameter of 2.13 nm, showing relatively high diffusivity (D = 0.5672 nm^2/s).

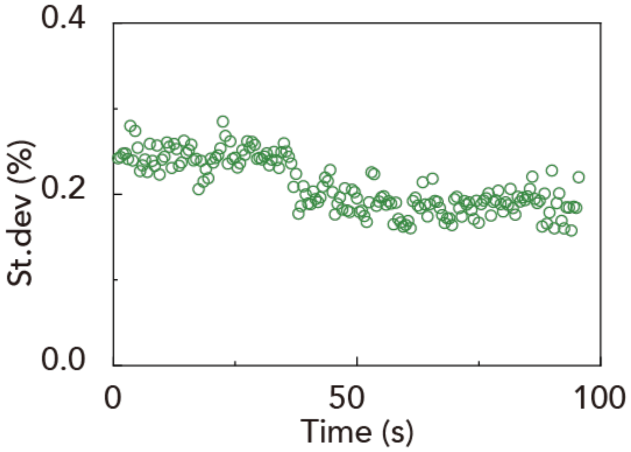

Supplemental Figure 2. Relative standard deviation changes of tracked Pt nanoparticles as a function of time. Related to Figure 2.

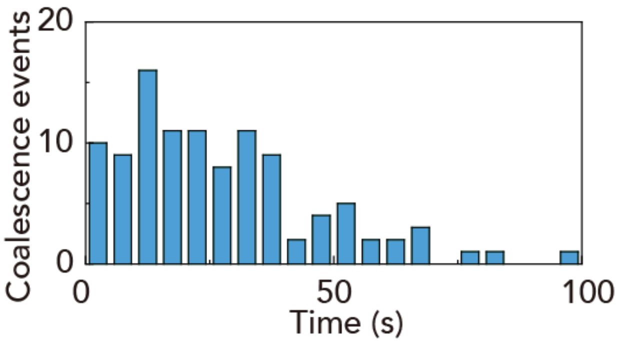

Supplemental Figure 3. Histogram of the number of the coalescence events by time in Movie S1. Related to Figure 2.

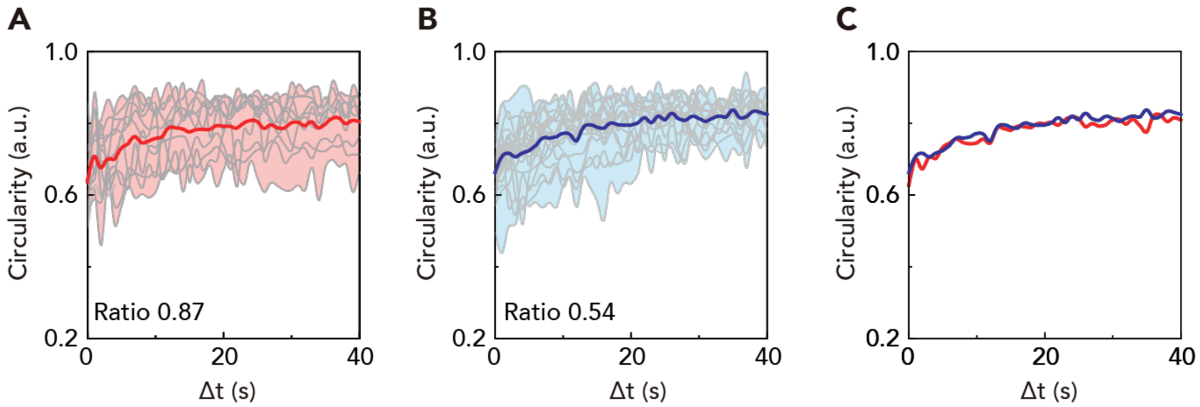

Supplemental Figure 4. Circularity change comparison between symmetric and asymmetric coalescence. Related to Figure 4.

(A) Overlap of tracked circularity for merged Pt nanoparticles with symmetric coalescence. The average ratio between coalescence pairs is 0.87.

(B) Overlap of tracked circularity for merged Pt nanoparticles with asymmetric coalescence. The average ratio between coalescence pairs is 0.54.

(C) Difference between symmetric & asymmetric coalescence in circularity change.

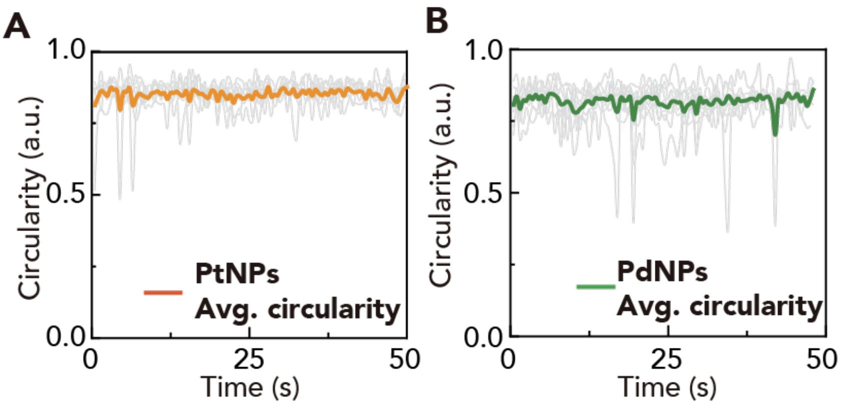

Supplemental Figure 5. Circularity change of the Pt and Pd nanoparticles without undergoing coalescence event. Related to Figure 4.

(A) Overlap of tracked circularity for merged Pt nanoparticles without coalescence during the observation time.

(B) Overlap of tracked circularity for merged Pd nanoparticles without coalescence during the observation time.

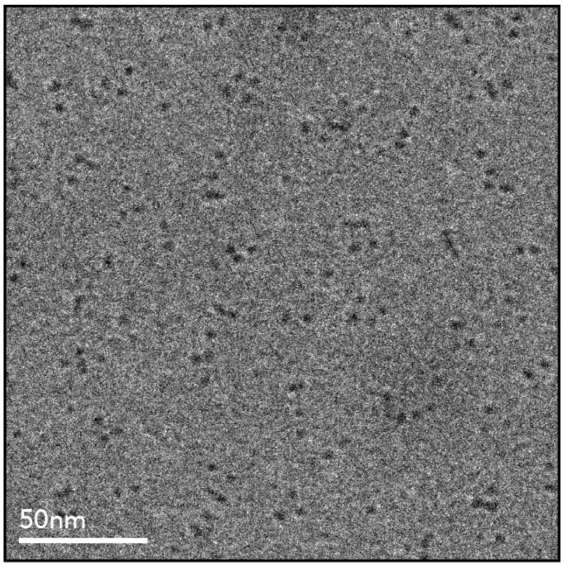

Supplemental Figure 6. TEM snap-shot image of Pd nanoparticle growth from Movie S5 at dose rate 100 $e^{-}Å^{-2}s^{-1}$. Related to Figure 4.

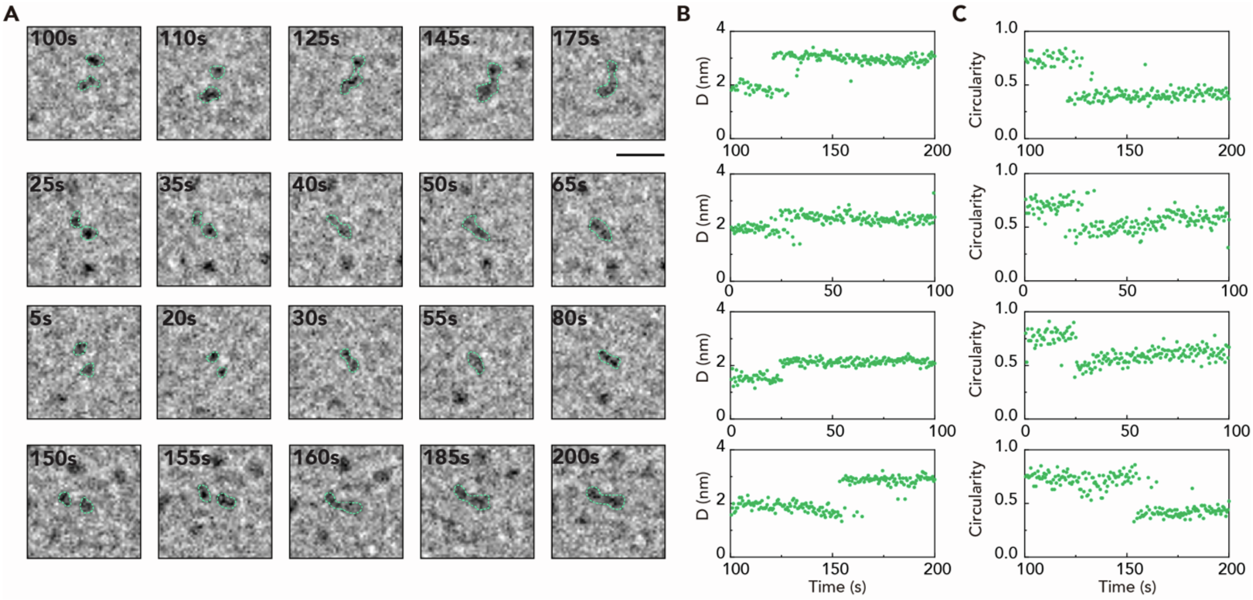

Supplemental Figure 7. Coalescence dynamics of Pd nanoparticles in Movie S5 at dose rate 100 $e^{-}Å^{-2}s^{-1}$. Related to Figure 4.

(A) TEM snap-shot images from MovieS5 for representative Pd nanoparticles that undergo coalescence growth pathways. Scale bar: 10nm.

(B) Size change of the nanoparticle shown in panel (A).

(C) Circularity change of the nanoparticles shown in panel (A).

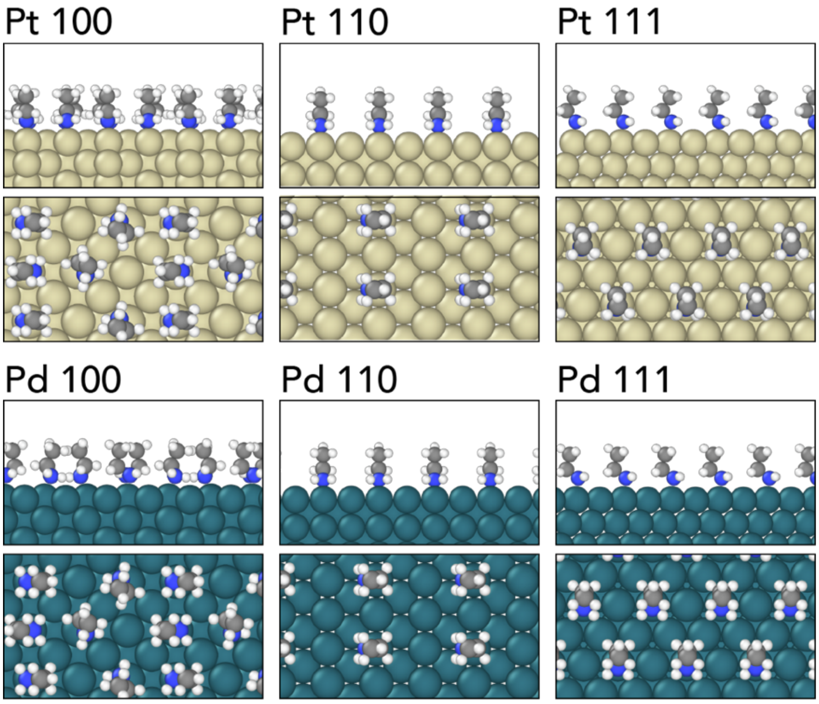

Supplemental Figure 8. Result of ligand binding conformation of Pt and Pd surface calculated by density functional theory. Related to Methods.

	C_f	C(t0)	k
Pt	0.799	0.637	0.315
Pd	0.739	0.465	0.154

Supplemental Table 1. Fitted parameter for circularity change in Figure 4C. Related to Figure 4.
